# High Performance of Al-MCM-41 in Tetracycline Adsorption Using a Fixed-Bed Column

**DOI:** 10.1021/acsomega.5c09936

**Published:** 2026-01-06

**Authors:** Henrique B. Gomes, José A. Giusti Zacharias, Laura P. Tovar, Sarah I. P. M. N. Alves, Luís A. M. Ruotolo, Romilda Fernandez-Felisbino

**Affiliations:** † Department of Chemical Engineering, 28105 Federal University of São Paulo, Diadema, São Paulo 09972-270, Brazil; ‡ Department of Physics, Federal University of São Paulo, Diadema, São Paulo 09972-270, Brazil; § Department of Chemical Engineering, 67828 Federal University of São Carlos, São Carlos, São Paulo 13565-905, Brazil

## Abstract

This study investigates the adsorption efficacy of aluminum-modified mesoporous silica (Al-MCM-41, Si/Al = 16.1) for the removal of tetracycline (TC) from aqueous solutions, employing both batch and fixed-bed column systems in the quest for sustainable solutions to eliminate emerging contaminants. The synthesized material exhibited a highly ordered hexagonal structure and high surface area (∼850 m^2^ g^–1^), confirmed by X-ray diffraction, nitrogen physisorption, FTIR, and transmission electron microscopy analyses. Batch adsorption kinetics followed a pseudo-second-order model, indicating surface-controlled uptake, while isotherm data fit both Langmuir and Freundlich models, with a maximum adsorption capacity of 82.74 mg g^–1^. Thermodynamic analysis revealed a spontaneous and exothermic process (Δ*H*° = −25.8 kJ mol^–1^), suggesting combined physisorption and weak chemisorption. In fixed-bed column experiments, the Yan model provided the best fit to breakthrough curves, achieving an adsorption capacity of 19.1 mg g^–1^ at an influent concentration of 15 mg L^–1^. These results demonstrate the good efficiency and practical applicability of Al-MCM-41 in continuous-flow systems for removing emerging contaminants such as TC, supporting the development of sustainable water treatment technologies.

## Graphical abstract

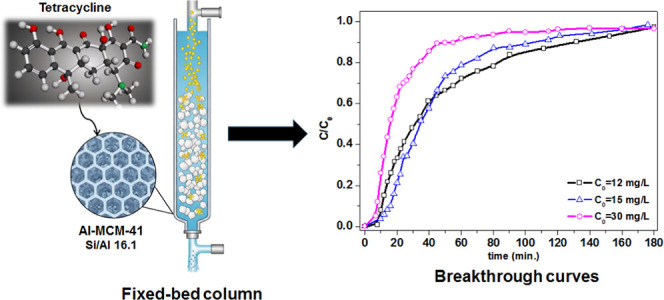

## 1 Introduction

Tetracycline (TC) is a broad-spectrum antibiotic widely used in both veterinary and human medicine, due to its broad spectrum of activity against Gram-positive and Gram-negative bacteria and protozoan parasites, among others, together with its minimal side effects.^1^ However, despite its therapeutic efficacy, TC is incompletely absorbed, with only 20–50% being metabolized by the body, while the remainder is excreted and released into the environment as an emerging contaminant (EC).^2,3^ The exposure of humans to antibiotics such as TC, due to contact with contaminated water and food, can lead to the development of antibiotic-resistant bacteria and genes (ARBs/ARGs), which, in the case of TC, can occur at low concentrations in the range 0.1–1.0 mg TC kg^–1^.^1,4^ Research undertaken in 2016 indicated that antibiotic resistance had already led to over 700,000 deaths worldwide, with estimates suggesting that by 2050, the number could reach 10 million.^5,6^ Meanwhile, the dependence of contemporary society on antibiotics such as TC means that their use is unlikely to cease in the foreseeable future. The production of these compounds is predicted to continue increasing exponentially in the next decades, inevitably leading to increased release of antibiotic residues in effluents.^6–8^

The development of sustainable and economically viable water treatment technologies for the removal of ECs is essential to avoid the harmful effects of these residues on the environment and human health. This highlights the interdependence among environmental, social, and economic dimensions, aiming to achieve the Agenda 2030 of Sustainable Development Goals of the United Nations.^4^ The challenge concerning ECs such as TC requires an integrated and multidisciplinary approach that addresses the need to both control the release of these substances into the environment and develop effective technologies for their removal.

In recent years, the removal of TC from aquatic systems has been investigated using physical, chemical, and biological processes.^2,9,10^ Disadvantages of many of these methods include high cost, generation of large quantities of solid waste, low applicability to different types of pollutants, high demands in terms of area and energy, and complex operation.^2,10,11^

The adsorption process is one of the EC removal methods that has attracted strong interest, due to its potential to remove different types of pollutants, including organic and inorganic compounds, from industrial effluents.^3^ This method can be applied to wastewater, underground water, surface water, and drinking water, offering the advantages of an attractive cost–benefit ratio, flexible design, low energy consumption, easy regeneration of the adsorbent, simple operation, versatility, and low sensitivity to chemical products.^3,12^

Adsorption involves the transfer of adsorbate molecules from the liquid phase to the surface of the solid adsorbent. Many adsorbents have been investigated for the removal of TC, among the most studied adsorbents, such as biosorbents, to more innovative materials, such as nanomaterials and polymers.^13–18^

Biosorption refers to processes employing biomass as biosorbents, including algae, bacteria, and fungi, which can offer advantages in terms of cost and availability of the material.^19–21^ However, the use of biomass for biosorbent production may be limited by regional and seasonal factors that can hinder their large-scale application. Therefore, in many situations, the effective removal of TC may require the use of synthetic adsorbents developed to provide high adsorption capacity, selectivity, and stability with the ability to control their physicochemical properties and adsorption performance. However, there are few reports in the literature concerning the application of MCM-41 silica modified with Al^3+^, obtained by hydrothermal synthesis, in the removal of TC in fixed-bed columns.^22–24^ In our previous study, the high potential of Al-MCM-41 for amoxicillin (AMX) removal was demonstrated, so the optimized material with a Si/Al ratio of 10.5 (∼760 m^2^ g^–1^) demonstrated an exceptional adsorption capacity of 132.2 mg AMX per gram of adsorbent, significantly exceeding that of other materials reported in the literature.^25^ This performance is the result of a balanced composition that maximizes the surface area and density of active aluminum adsorption sites. In this sense, it is believed that the Al-MCM-41 sample (Si/Al = 16.1), with its larger surface area (≈850 m^2^ g^–1^) and the contribution of framework aluminum, may exhibit strong potential for tetracycline adsorption.^25^

This study aims to evaluate the efficiency of Al-MCM-41 (Si/Al, 16.1) as an adsorbent for TC removal in fixed-bed columns, focusing on the adsorption capacity and breakthrough curve analysis. In batch, the kinetics, isotherms, and thermodynamic parameters of adsorption of TC were also investigated. A laboratory-scale and fixed-bed column system was used with calcined Al-MCM-41 to determine the effects of the initial TC concentration on the breakthrough and saturation times (*t*_b_ and *t*_s_), maximum adsorption capacity (*q*_exp_), fractional bed utilization (FBU), and mass-transfer zone (MTZ). By addressing these objectives, this study not only contributes to the field of wastewater treatment but also lays the groundwork for future research on sustainable and efficient removal of antibiotics from effluents.

## 2 Experimental Section

### 2.1 Preparation of Al-MCM-41

Mesoporous Al-MCM-41 silica was prepared according to a previously described method, with modifications.^24,25^ Briefly, cetyltrimethylammonium bromide (CTMABr, CH_3_(CH_2_)_15_N­(CH_3_)_3_Br, ≥98%, Sigma-Aldrich) and tetraethyl orthosilicate (TEOS, Si­(OC_2_H_5_)_4_, 98%, Sigma-Aldrich) were dissolved in an alkali solution (NaOH, 97%, Synth) and mixed using a magnetic stirrer for 30 min at room temperature to obtain a homogeneous solution. An aqueous solution of sodium aluminate (NaAlO_2_, ≥99.95%, Sigma-Aldrich) was then added to the solution to obtain a Si/Al ratio of 16.1 (determined in a previous work). The mixture was stirred vigorously for 120 min at room temperature, followed by increasing the temperature to 80 °C and continuous stirring until the solution was thoroughly homogenized. After being aged for 20 min, the gel material was allowed to cool naturally to room temperature and then maintained under magnetic agitation for an additional 4 h. All of the chemical reagents used in this study were used as received. Aqueous solutions were prepared using deionized water from a Milli-Q system (18.0 MΩ cm resistivity at 25 °C) and stored at 4 °C. The gel obtained had the following molar composition

$${SiO}_{2}:0.03{Al}_{2}O_{3}:0.27{Na}_{2}O:0.089CTMABr:130.0H_{2}O$$

The gel was transferred to a 125 mL Teflon-lined stainless steel autoclave, and a hydrothermal process was performed at 150 °C for 12 h. The resulting solid powder was filtered and washed several times with distilled water to obtain a neutral filtrate. Finally, the solid was dried overnight (12 h) in an oven at 120 °C. To remove the CTMABr surfactant, the obtained powder was transferred to a ceramic crucible and calcined at 550 °C for 5 h in air at a ramping rate of 2 °C min^–1^. The resulting material, labeled as Al-MCM-41 adsorbent, was used in the adsorption experiments.

### 2.2 Characterization of the Al-MCM-41 Adsorbent

Different analytical techniques were employed to characterize the structure and properties of the adsorbents before and after TC adsorption. Low-angle powder X-ray diffraction measurements enabled a quantitative evaluation of the crystal structure of Al-MCM-41. The diffractograms were acquired using a Bruker D8 Advance instrument operating with Cu Kα radiation (λ = 1.54 Å), in the 2θ range 1–10°, with scanning at a rate of 0.5° min^–1^. Al-MCM-41 was characterized by the nitrogen adsorption isotherm method at 77 K, in the relative pressure range of 0.06–0.97, using a NOVA 1200 analyzer (Quantachrome Instruments). The particle size and morphology of the material were evaluated using transmission electron microscopy (TEM) at 100 kV. The amount and strength of the acid sites of Al-MCM-41 were determined by *n*-butylamine potentiometric titration using the MARCONI potentiometer model PA200, with a glass electrode; 50 mg of Al-MCM-41 was suspended in CH_3_CN and magnetically stirred by 8 h. This sample was titrated with a slow addition of portions of *n*-butylamine (100 μL, 0.025 mol L^–1^) until that electrode potential remained stable after the addition of the base.^26,27^ The functional groups present on Al-MCM-41 were investigated by Fourier transform infrared spectroscopy using an IR Prestige-21 spectrometer (Shimadzu) operating in transmittance mode in the 4000–400 cm^–1^ region. The samples were dried at 100 °C for 12 h and stored in a desiccator to avoid water adsorption. To acquire infrared spectra, the samples (0.1%) were mixed with KBr and pressed into tablets.

### 2.3 Tetracycline Adsorption Batch Experiments

The stock solutions of TC (C_22_H_24_N_2_O_8_·HCl, obtained from Prati-Donaduzzi) were prepared by dissolving a pharmaceutical capsule containing 500 mg of the antibiotic. The kinetic and equilibrium behaviors of TC adsorption by Al-MCM-41 (Si/Al = 16.1) in an aqueous KH_2_PO_4_ solution (0.01 M) were studied using Erlenmeyer flasks (a batch system) at 25 °C. The as-prepared sample (adsorbent) was uniformly placed in a TC solution at a solid-to-liquid ratio of 1:1. In the adsorption kinetic experiments performed at pH 7.0, the known initial concentrations of TC were 30 and 100 mg L^–1^, with aliquots removed at 5, 10, 15, 20, 25, 40, 60, and 90 min. An adsorption isotherm study was performed using TC concentrations of 10, 20, 30, 40, 50, 60, 70, 80, 90, and 100 mg L^–1^ under continuous stirring for 90 min at 250 rpm with an orbital shaker. After the contact time, the TC/adsorbent aqueous suspensions were centrifuged at 4000 rpm for 60 min and filtered through 0.22 μm microporous syringe filters. The filtered solutions were analyzed using a UV–vis spectrophotometer (model HP 8453, Hewlett-Packard) at a TC maximum absorption wavelength of 360 nm to determine the adsorption equilibrium concentration of TC. The spectrophotometer was precalibrated with TC in the concentration range of 3–30 mg L^–1^. The TC adsorption capacity of the adsorbent (*q*_e_, mg g^–1^) was calculated using eq 1.

1 $$q_{e}=V\left(\frac{C_{0}-C_{e}}{W}\right)$$

where *C*_0_ (mg L^–1^) and *C*_e_ (mg L^–1^) are the initial and equilibrium concentrations of TC, respectively; *W* (g) is the mass of the adsorbent; and *V* (L) is the TC solution volume. The equilibrium adsorption isotherms were assessed using the *C*_e_ and *q*_e_ values, applying the Langmuir and Freundlich nonlinear adsorption models given by eqs 2 and 3, respectively.^28,29^

2 $$q_{e}=\frac{q_{\max}K_{L}C_{e}}{1+K_{L}C_{e}}$$

3 $$q_{e}=K_{f}C_{e}^{1/n}$$

where *q*_max_ (mg g^–1^) and *K*_L_ (L mg^–1^) are the maximum adsorption capacities of Al-MCM-41 and the Langmuir isotherm constant, respectively. The constants of the Freundlich isotherm model are *K*_F_ (mg g^–1^) and 1/*n* (L mg^–1^), which are related to the affinity of the adsorbate for the solid surface. The adsorption capacity at time *t* (*q*_*t*_, mg g^–1^) was evaluated using the pseudo-first-order and pseudo-second-order nonlinear models, described by eqs 4 and 5, respectively.^30,31^

4 $$q_{t}=q_{e}\left(1-e^{k_{1}t}\right)$$

5 $$q_{t}=\frac{k_{2}q_{e}^{2}t}{1+k_{2}q_{e}t}$$

where *q*_e_ and *q*_*t*_ are the adsorption capacities (mg g^–1^) at equilibrium and time *t* (min), respectively, and *k*_1_ (min^–1^) and *k*_2_ (g mg^–1^ min^–1^) are the adsorption rate constants for the pseudo-first-order and pseudo-second-order models, respectively.

### 2.4 Fixed-Bed Column Adsorption Experiments

A schematic diagram of the laboratory-scale fixed-bed adsorption column (with the zoom of the filled column photo, self-authored) is shown in Figure 1.

**Figure 1:**
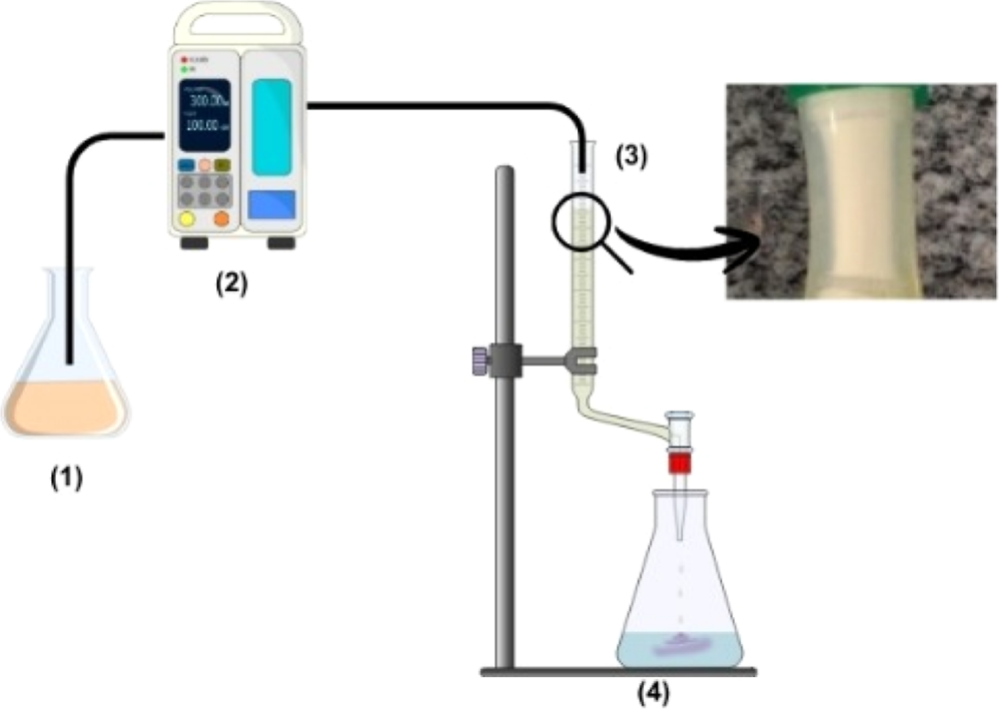
Schematic diagram of the fixed-bed adsorption column: (1) TC solution; (2) peristaltic pump; (3) bed with adsorbent; and (4) effluent and zoom of the filled column photo—self-authored.

The adsorption of TC onto Al-MCM-41 (Si/Al = 16.1) was performed using a silicone adsorption column (5 mm i.d. × 20 mm length) randomly filled with Al-MCM-41 to a bed height of 15 mm. TC adsorption assays were performed under isothermal conditions at 25 °C using three inlet TC concentrations (12, 15, and 30 mg L^–1^). The TC solutions, contained in borosilicate glass bottles (250 mL), were delivered by using a peristaltic pump (model BP 600, Milan) set at a flow rate of 1.45 mL min^–1^. The liquid residence time is approximately 180 min.

The TC concentration in the effluent solution was monitored by using UV–vis spectrophotometry at a wavelength of 360 nm. The breakthrough time (*t*_b_) was determined from the breakthrough curve at *C*_b_/*C*_0_ = 0.05, whereas the saturation time (*t*_s_) corresponded to *C*_s_/*C*_0_ = 0.95.

6 $$q_{\exp}=\frac{Q}{W}\int_{0}^{t}\left(C_{0}-C_{t}\right)\;dt$$

7 $$q_{b}=\frac{C_{0}Q}{W}\int_{0}^{t_{b}}\left(1-\frac{C_{b}}{C_{0}}\right)$$

8 $$q_{s}=\frac{C_{0}Q}{W}\int_{0}^{t_{s}}\left(1-\frac{C_{s}}{C_{0}}\right)$$

9 $$FBU=\frac{q_{b}}{q_{s}}$$

10 $$MTZ=Z\left(1-\frac{t_{b}}{t_{s}}\right)$$

In the above equations, *C*_0_, *C*_b_, and *C*_s_ are the initial, breakthrough, and saturation concentrations of TC (mg L^–1^), respectively; *t*_b_ and *t*_s_ are the breakthrough and saturation times (min), respectively; *Q* is the flow rate (1.45 mL min^–1^); *W* is the weight of the adsorbent in the column for *C*_0_: (a) 12 mg L^–1^, *W* = 0.0553 g, (b) 15 mg L^–1^, *W* = 0.0561 g, and (c) 30 mg L^–1^, *W* = 0.0560 g; *q*_exp_, *q*_b_, and *q*_s_ are the experimental breakthrough and saturation amounts of adsorbed TC (mg g^–1^), respectively; FBU is the fractional bed utilization, and MTZ is the mass-transfer zone (mm) using bed height *Z* = 15 mm.

The nonlinear Bohart–Adams,^32^ Yan,^33^ and Clark^34^ models were applied to model the dynamics of TC adsorption in a fixed-bed column (eqs 11–14). The parameters used to evaluate the quality of fit of the models were the coefficient of determination (*R* ^2^) and the root-mean-square error (RMSE).

11 $$\frac{C}{C_{0}}=\exp\left(k_{BA}C_{0}t-\frac{k_{BA}N_{0}Z}{\upsilon }\right)$$

12 $$\upsilon =\frac{Q}{A}$$

13 $$\frac{C}{C_{0}}=1-\frac{1}{1+{\left(\frac{C_{0}Qt}{{q_{Y}}^{m}}\right)}^{a_{Y}}}$$

14 $$\frac{C_{t}}{C_{0}}=\frac{1}{{\left(1+A{\;e}^{-rt}\right)}^{1/\left(n-1\right)}}$$

In the above equations, *k*_BA_ is the Bohart–Adams model rate constant (mL min^–1^ mg^–1^); *N*_0_ is the uptake volumetric capacity (mg L^–1^); *Z* is the bed height (cm); υ is the linear flow rate (cm min^–1^); *A* is the column cross-sectional area (cm^2^); *q*_Y_ is the amount of solute adsorbed (mg g^–1^); *a*_Y_ is the constant of the Yan model; *n* (dimensionless) is the Freundlich isotherm model constant; and *A* (dimensionless) and *r* (1/min) are the constants of the Clark model.

## 3 Results and Discussion

### 3.1 Characterization of the Al-MCM-41 Adsorbent

The X-ray diffractograms of the as-synthesized and calcined Al-MCM-41 (16.1) samples (Figure 2A) exhibited three typical peaks characteristic of the hexagonal structure of MCM-41, with one attributed to the (100) plane and the other two of lower intensity. A significant increase in intensity was observed for the calcined solid, where the increase in intensity related to the (100) plane in the Al-MCM-41C diffractogram indicating contraction of the lattice caused by the removal of the surfactant from within the mesoporous and condensation of silanol groups.^35^ The attenuation of the diffraction signal in the presence of the surfactant was due to the difference in electron density between the pore walls (silica/alumina) and the pore interior (filled with surfactant).^36^

**Figure 2:**
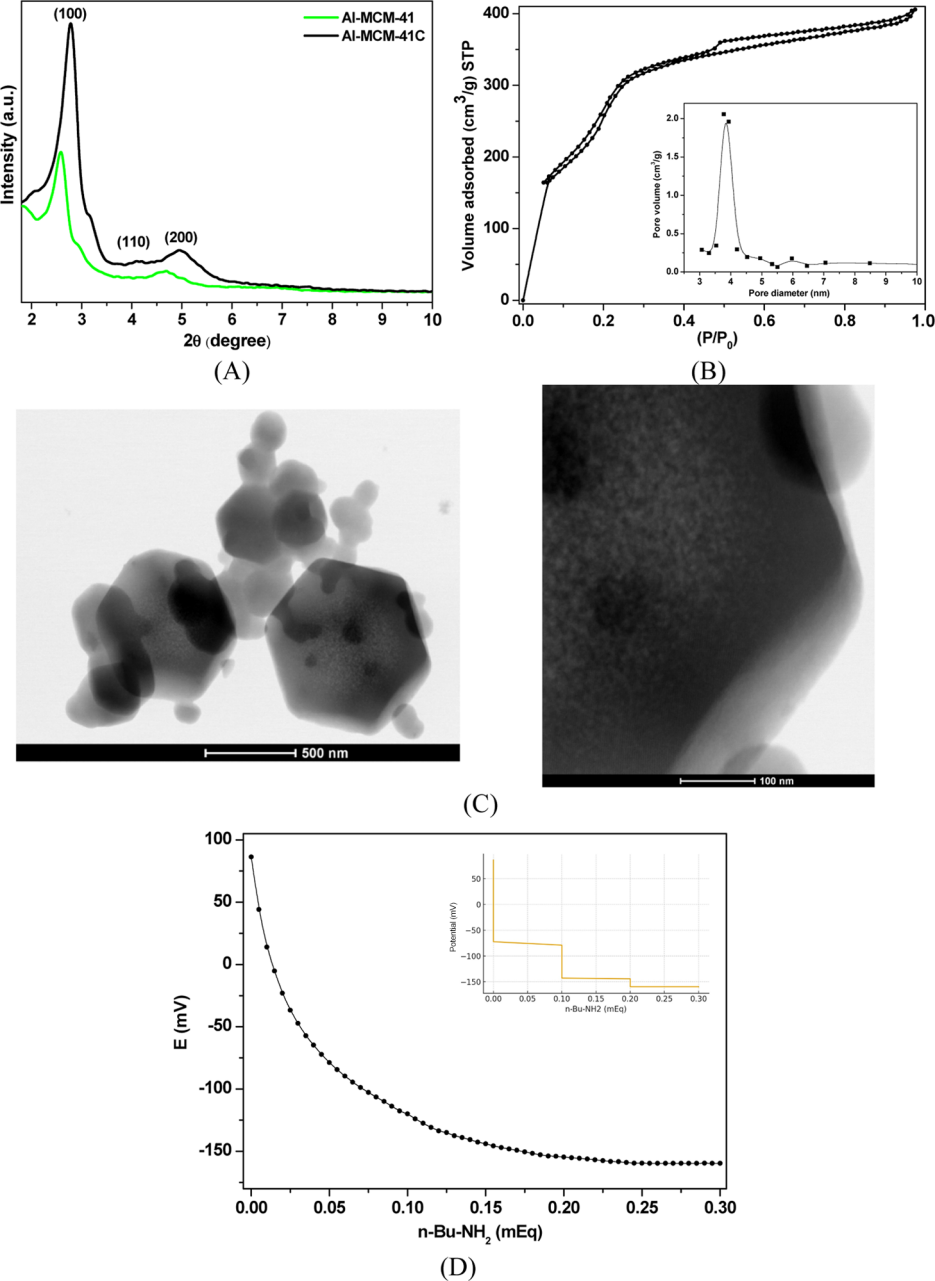
Characterization of Al-MCM-41: (A) X-ray diffractograms, (B) N_2_ adsorption/desorption isotherms; inset, BJH pore size distribution curve, (C) TEM images and, (D) curve of potentiometric titration of Al-MCM-41.

The N_2_ adsorption/desorption isotherms of the Al-MCM-41 adsorbent (Figure 2B) were characteristic of type IV, with a slight hysteresis in the *P*/*P*_0_ range from 0.30 to 0.45, indicative of capillary condensation in uniform mesopores.^22,23,37^ The results for the textural parameters showed that the Al-MCM-41 sample containing aluminum presented a BET specific area of approximately 850 m^2^ g^–1^, and the BJH mean pore diameter is 3.88 nm. The pore size distribution of Al-MCM-41 is presented in the inset of Figure 2B; it verified that the pores of 4 nm diameter occupied most part of the pore volume of this sample, and it showed that the pores of the sample are very uniform, typical of highly ordered MCM-41 silica.^35,37^ These results indicated that the insertion of a low Al content (Si/Al = 16.1) into the MCM-41 structure did not alter the textural properties, with no effect on the ordering of the silica hexagonal structure, in agreement with the TEM images of as-synthesized Al-MCM-41 (Figure 2C). Compared to Al-MCM-41 synthesized by Lin et al. (2021), our adsorbent exhibits a higher surface area (850 m^2^ g^–1^) which may contribute to its superior adsorption capacity for tetracycline.^37^ However, when compared with the Al-MCM-41 samples (Si/Al = 24) obtained by Cesteros and Haller (2001), a lower degree of ordering is observed. This is due to the lower Al content of the sample but mainly to the synthesis method adopted by the authors.^35^ Figure 2D shows the potentiometric titration results; the acid strength of acid sites of the Al-MCM-41 sample can be classified as follows: *E*_i_ > 100 mV (very strong sites), 0 < *E*_i_ < 100 mV (strong sites), −100 < *E*_i_ < 0 (weak sites), and *E*_i_ < −100 mV (very weak sites).^26^ This result indicates that the Al-MCM-41 adsorbent exhibits a combination of strong, intermediate, and weak acid sites with a predominance of medium-strength acidity. This behavior is attributed to Al^3+^ incorporated into the framework, generating Brønsted sites of moderate intensity, along with an additional amount of weak sites originating from silanol groups.^27^

### 3.2 Adsorption Kinetics, Equilibrium Isotherms, and Thermodynamic Parameters

Figure 3B shows the results of the amount of TC adsorbed by Si-MCM-41 and Al-MCM-41 as functions of the contact time. It is observed that the solid without aluminum (Si-MCM-41) exhibits a negligible adsorption capacity when compared with the Al-MCM-41 adsorbent. The kinetic data (Figure 3) indicated that the adsorbate (TC) molecules had a high affinity for the Al-MCM-41 surface, with a very rapid adsorption for *C*_0_ of both 30 and 100 mg L^–1^, reaching the adsorption equilibrium after approximately 60 min of contact. Increasing *C*_0_ from 30 mg L^–1^ (Figure 3A) to 100 mg L^–1^ (Figure 3B) resulted in the adsorption capacity increasing from 15.38 to 54.80 mg g^–1^, due to the higher mass-transfer driving force of the adsorbate from the bulk solution to the adsorbent surface.^38,39^

**Figure 3:**
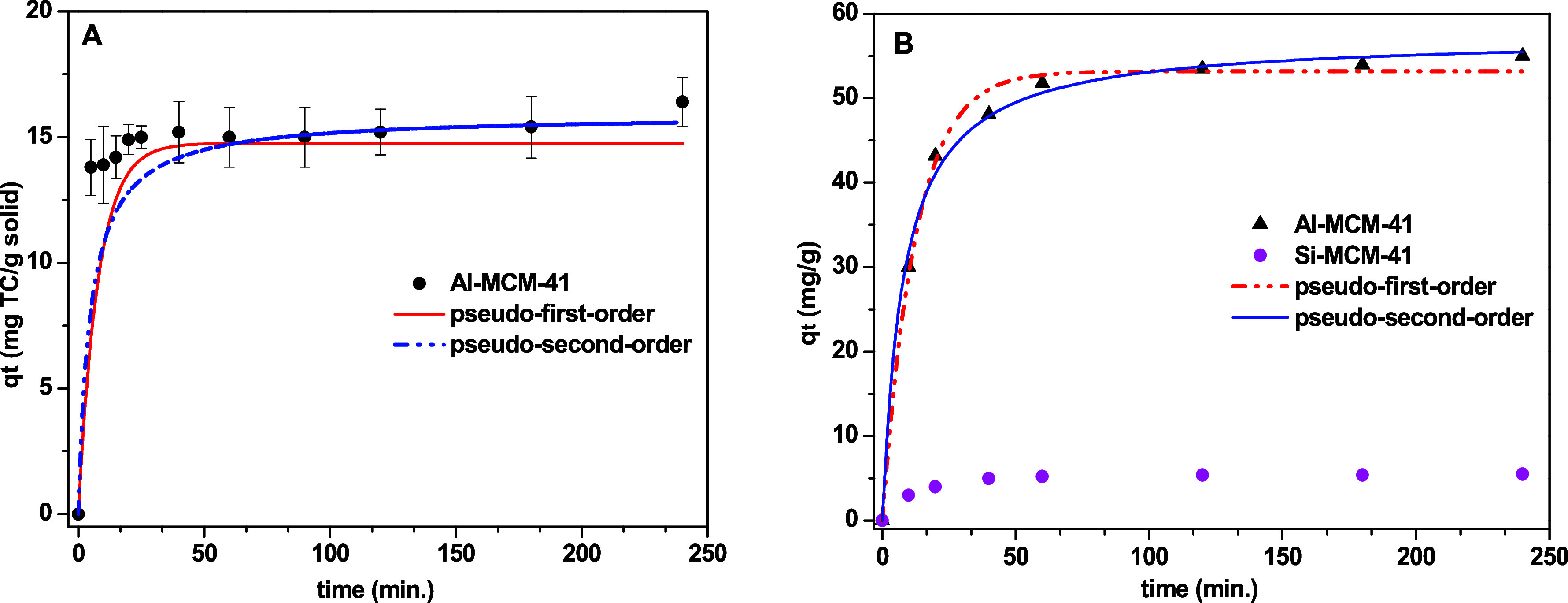
Tetracycline adsorption at pH 7.0, 25 °C, and nonlinear kinetic curves: (A) Al-MCM-41 with *C*_0_ = 30 mg L^–1^ and (B) Si-MCM-41 and Al-MCM-41 with 100 mg L^–1^.

The fits of the nonlinear models are shown in Figure 3, and the corresponding parameter values and coefficients of determination are listed in Table 1. Higher *R* ^2^ values were obtained for the pseudo-second-order model (0.989–0.996), compared to the pseudo-first-order model (0.971–0.985), showing that the adsorption kinetics data were better described by the pseudo-second-order model.^16,39^ These results were consistent with the studies reported in the literature and suggested that the adsorption is controlled by a chemical process, with the adsorption rate being proportional to the number of active sites on the adsorbent surface.^20,21,39^

**Table 1:** Parameters and Determination Coefficients of the Pseudo-First-Order and Pseudo-Second-Order Models Applied to the Adsorption Kinetics Data Obtained at pH 7.0 and 25 °C

*C*_0_ (mg L^–1^)	pseudo-first-order		pseudo-second-order	
30	*q*_1_	15.05 mg/g	*q*_2_	15.38 mg/g
	*k*_1_	0.936 min^–1^	*k*_2_	0.172 min^–1^
	*R* ^2^	0.982	*R* ^2^	0.990
100	*q*_1_	60.00 mg/g	*q*_2_	54.80 mg/g
	*k*_1_	0.002 min^–1^	*k*_2_	0.081 min^–1^
	*R* ^2^	0.983	*R* ^2^	0.991

The TC adsorption equilibrium data were fitted by using the classical Langmuir and Freundlich models (Figure 4), enabling inferences to be made regarding the behavior of the system and the characteristics of the Al-MCM-41 adsorbent surface.

**Figure 4:**
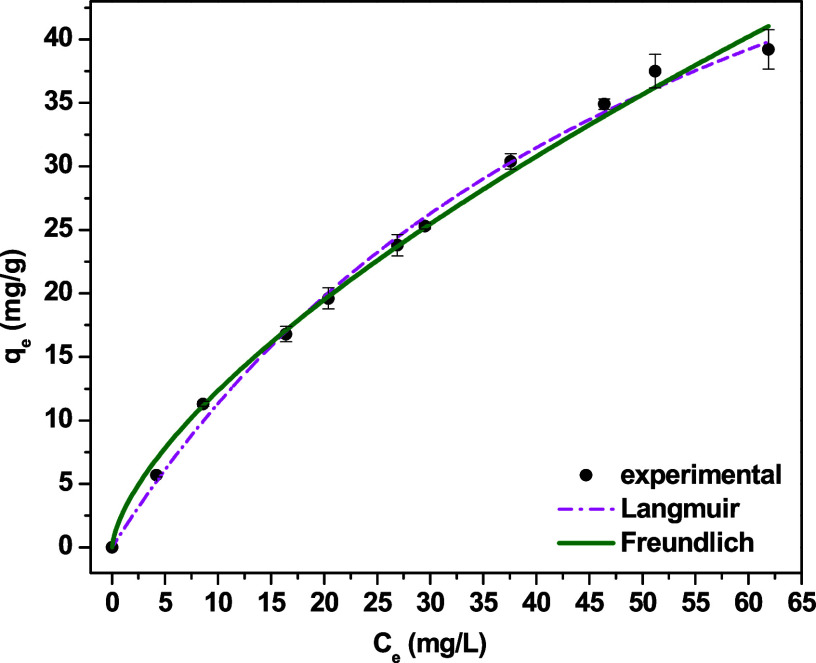
Adsorption isotherm for TC on Al-MCM-41 and nonlinear fits of the Langmuir and Freundlich models (*C*_0_ = 10–100 mg L^–1^, pH 7.0, 25 °C, 90 min).

Both models provide a good fit to the experimental data, with similar *R* ^2^ values of 0.980 and 0.985 for the Langmuir and Freundlich models, respectively.

The results (Table 2) showed a high maximum adsorption capacity (82.74 mg g^–1^) which was comparable or superior to the values for many adsorbents reported in the literature, demonstrating the potential of the Al-MCM-41 adsorbent for the removal of TC.^16,17^ The Langmuir constant (*K*_L_ = 0.015 L mg^–1^) indicated a moderate affinity for TC and the active sites of Al-MCM-41. However, the Freundlich model provided a better representation of the behavior of the experimental system, indicating the heterogeneous characteristics of the adsorbent surface, which was consistent with the presence of Al in the Si-MCM-41 lattice. The *n*_F_ parameter value (1.467) indicates favorable adsorption, suggesting that the adsorption process occurs easily at low concentrations, which is an important feature for the removal of emerging contaminants.

**Table 2:** Parameters and Determination Coefficients of the Nonlinear Langmuir and Freundlich Isotherm Models Applied to TC Adsorption Equilibrium Data^a^

Langmuir		Freundlich	
*q*_max_	82.74 mg/g	*K*_F_	2.491 (mg/g) (L/mg)^1/*n*^
*K*_L_	0.015 L/mg	*n*_F_	1.467
*R* ^2^	0.980	*R* ^2^	0.985

a *q*_max_: maximum adsorption capacity; *K*_L_: adsorbate/adsorbent interaction constant; *n*_F_: constant related to surface heterogeneity; *K*_F_: Freundlich adsorption capacity constant.

The good fit obtained with the Langmuir model indicated that despite the heterogeneity of the surface, there could have been specific sites on Al-MCM-41 with near-ideal behavior (monolayer), which was plausible given that the adsorbate was in solution. The interactions probably involve different simultaneous mechanisms, such as electrostatic interactions, hydrogen bonding, π–π interactions, and complexation with functional groups on the surface.^14,16,40^ The good adsorption performance of TC by Al-MCM-41 following this mechanism can be supported by the potentiometric titration results (Figure 2D). The coexistence of these different types of acid sites contributes to the material’s ability to efficiently interact with polar and zwitterionic molecules, such as tetracycline, favoring combined mechanisms of electrostatic interaction and hydrogen-bond formation.^14,16,27^

Overall, the results indicated that the Al-MCM-41 adsorbent presented good adsorption capacity when compared with the results reported in the literature (Table 3), making it a promising material for the removal of TC from aqueous effluents.

**Table 3:** Different Sorbents for TC Adsorption

adsorbent	pH	temperature (°C)	adsorption capacity (mg g^–1^)	reference
iron(III)-loaded CNFs	4	25	294.12	^13^
magnetic chitosan	7	20	211.21	^14^
shrimp shell waste	7	25	381.75	^15^
activated carbon	7	-	17.38	^16^
graphene oxide calcium alginate	6	25	131.6	^17^
SiO nanoparticles	5	23	552.48	^41^
copper carboxymethyl cellulose nanoparticles	7.5	25	0.666	^42^

The values obtained for the thermodynamic parameters (Table 4) showed that the adsorption process was spontaneous (Δ*G*° < 0) at lower temperatures, while the spontaneity decreased with increasing temperature, demonstrating that the process was exothermic.^14,39^ The negative Δ*H*° confirmed the exothermic nature of the process, while the negative Δ*S*° revealed that the disorder in the system decreased during the adsorption. This behavior was expected because the TC molecules left the aqueous phase and were organized on the surface of the adsorbent.

**Table 4:** Thermodynamic Parameters for the Adsorption of TC onto Al-MCM-41 (*C*_0_ = 30 mg L^–1^, pH 7.0, 90 min)

*q* (mg L^–1^)	*T* (K)	Δ*G*° (kJ mol^–1^)	Δ*H*° (kJ mol^–1^)	Δ*S*° (kJ mol^–1^ K^–1^)	*R* ^2^
18.6	308.15	–1.39	–25.8	–0.08	0.993
18.1	318.15	–0.57			
12.9	328.15	0.25			
12.6	338.15	1.06			

The relatively high Δ*H*° value (−25.8 kJ mol^–1^) is typical of physical adsorption (physisorption), with a possible contribution of chemical interactions (weak chemisorption). This result supported the hypothesis of the existence of specific interactions, such as hydrogen bonding, complexation, or electrostatic interactions, in the TC-Al-MCM-41 system.^14,39^

FTIR spectra can help describe the potential roles in adsorption and provide a more comprehensive understanding of the mechanisms involved and their interactions. Figure 5 shows the FTIR spectra obtained for the Al-MCM-41 adsorbent before and after TC adsorption. The broad band at ∼3450 cm^–1^, which could be attributed to the O–H stretching vibrations of the surface hydroxyl groups or adsorbed water molecules, increased after the adsorption of TC, indicating possible hydrogen bridge interactions between the −OH groups on the surface of Al-MCM-41 and TC functional groups such as −OH, −NH_2_, or =O. The presence of TC on the surface was evidenced by the increase of the band in the region 1650–1550 cm^–1^ related to C=O (carbonyl) and the amide vibrations of TC, suggesting chemical interactions between the carbonyl of the adsorbate and the acidic sites of Al-MCM-41.^12,15,18,39^

**Figure 5:**
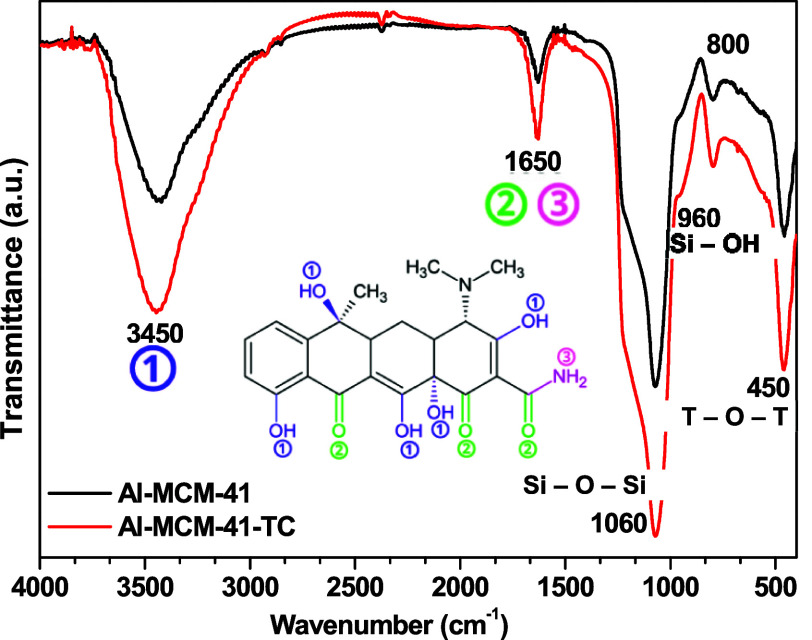
FTIR absorption spectra of Al-MCM-41, before and after the adsorption of TC.

The good adsorption capacity of TC exhibited by Al-MCM-41 is mainly due to the presence of Al in the silica framework. At pH 7.0, TC is predominantly in its zwitterionic form (with protonated amine groups and partially deprotonated phenolic and enolic groups), which enables simultaneous electrostatic interactions and hydrogen bonding. The −OH and −NH groups of TC can also form hydrogen bonds with silanol groups (Si–OH) and with hydroxyls adsorbed on the surface of Al-MCM-41, further contributing to the adsorption process. These changes can be observed in the FTIR spectra in Figure 6, where an increase in the bands assigned to carbonyl/N–H and −OH vibrations is seen.

**Figure 6:**
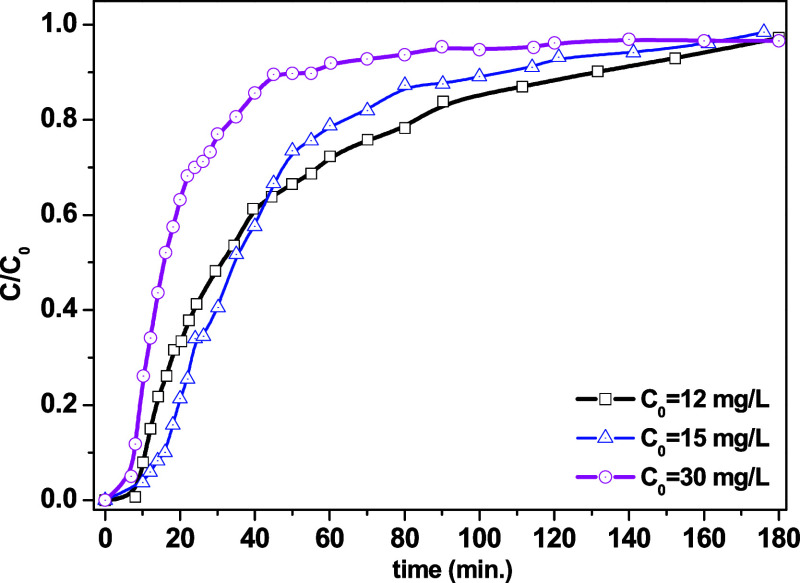
Breakthrough curves for TC adsorption onto Al-MCM-41 in a fixed-bed column and *Q* = 1.5 mL min^–1^, for *C*_0_:(i) 12 mg L^–1^, *W* = 0.0553 g, (ii) 15 mg L^–1^, *W* = 0.0561 g, and (iii) 30 mg L^–1^, *W* = 0.0560 g.

### 3.3 Column Adsorption Studies

Figure 6 shows the breakthrough curves obtained for *C*_0_ values of 12, 15, and 30 mg L^–1^. The curve profiles were typical of well-distributed mesoporous adsorbents, with near-ideal behavior.^43–45^

An increase in the initial concentration (*C*_0_) resulted in a shorter retention time, reflecting the rapid saturation of the column at higher tetracycline loading. The saturation time was also significantly reduced, as expected, since the deactivation of the adsorbent was faster at higher TC concentrations.^46^

Table 5 shows the experimental data for the operation of the column (*Q*, *C*_0_, and adsorbent mass) together with the calculated values for the breakthrough time (*t*_b_), saturation time (*t*_sat_), experimental adsorption capacity (*q*_exp_), and FBU. The *q*_exp_ values indicated an optimal column feed concentration (*C*_0_) of 15 mg L^–1^ at which the adsorption capacity was maximized, reflecting a higher load available for adsorption before the bed became saturated. The FBU increased from 0.16 to 0.29 with an increase in *C*_0_, indicating an improved relative utilization of the bed at higher concentrations. On the other hand, values <0.3 indicated that most of the bed only participated in the adsorption after the onset of breakthrough, showing that the process still required optimization.^46,47^ The results suggested that the service time of the column would decrease with increasing *C*_0_. Although higher concentrations are associated with a higher initial adsorption capacity, the operational stability of the column was reduced, which could affect continuous systems.

**Table 5:** Experimental Conditions and Performance Parameters for Fixed-Bed Column Operation

experimental conditions				parameters				
*C*_0_ (mg/L)	*Q* (mL/min)	*V*_C_ (cm^3^)	ε	*t*_b_ (min)	*t*_sat_ (min)	*q*_exp_ (mg/g)	FBU	MTZ (mm)
12	1.5	0.39	0.4	9.2	150	15.7	0.16	14.1
15				10.9	140	19.1	0.25	13.8
30				6.9	90	18.9	0.29	13.8

It is worth to mention that the values of *q*_exp_ were coherent with the values predicted from the isotherm considering the initial tetracycline concentration used in the column experiments (for *C*_0_ = 12 mg L^–1^, 15 mg L^–1^, and 30 mg L^–1^, the values of *q* determined from the isotherm (Figure 5) were 12.5 mg g^–1^, 15.7 mg g^–1^, and 25 mg g^–1^), with the largest deviation observed at the highest concentration, which can be attributed to the sharp MTZ, indicating that adsorption sites were not effectively used for adsorption.

Considering *C*_0_ of 15 mg TC L^–1^, an experimental adsorption capacity of 19.1 mg g^–1^ was obtained. This high adsorption capacity along with FBU of 0.25 indicates high efficiency for practical applications. These results suggested that the optimal operation of the column depends on the balance between the adsorption capacity and service time of the bed.^48^ The MTZ occupied a very large fraction of the bed: 14.1 mm (93.9%) for *C*_0_ = 12 mg L^–1^, 13.8 mm (92.0%) for *C*_0_ = 15 mg L^–1^, and 13.8 mm (92.0%) for *C*_0_ = 30 mg L^–1^. These results indicate that, under the tested conditions, the adsorption front develops along almost the entire bed height, reducing the effective utilization of the column and suggesting a regime controlled by mass-transfer resistances (intraparticle diffusion and/or axial dispersion). For practical applications and scale-up design, it is recommended to increase the bed height (initial estimate ≈50 mm to reduce the MTZ to ≈30% of the bed) or operate at lower flow rates.^43–46^

Figure 7 shows the experimental breakthrough curves fitted to the nonlinear Bohart–Adams, Clark, and Yan models.^32–34^ All of the models provided satisfactory fits to the experimental data, with typical behavior for adsorption using a fixed-bed column.

**Figure 7:**
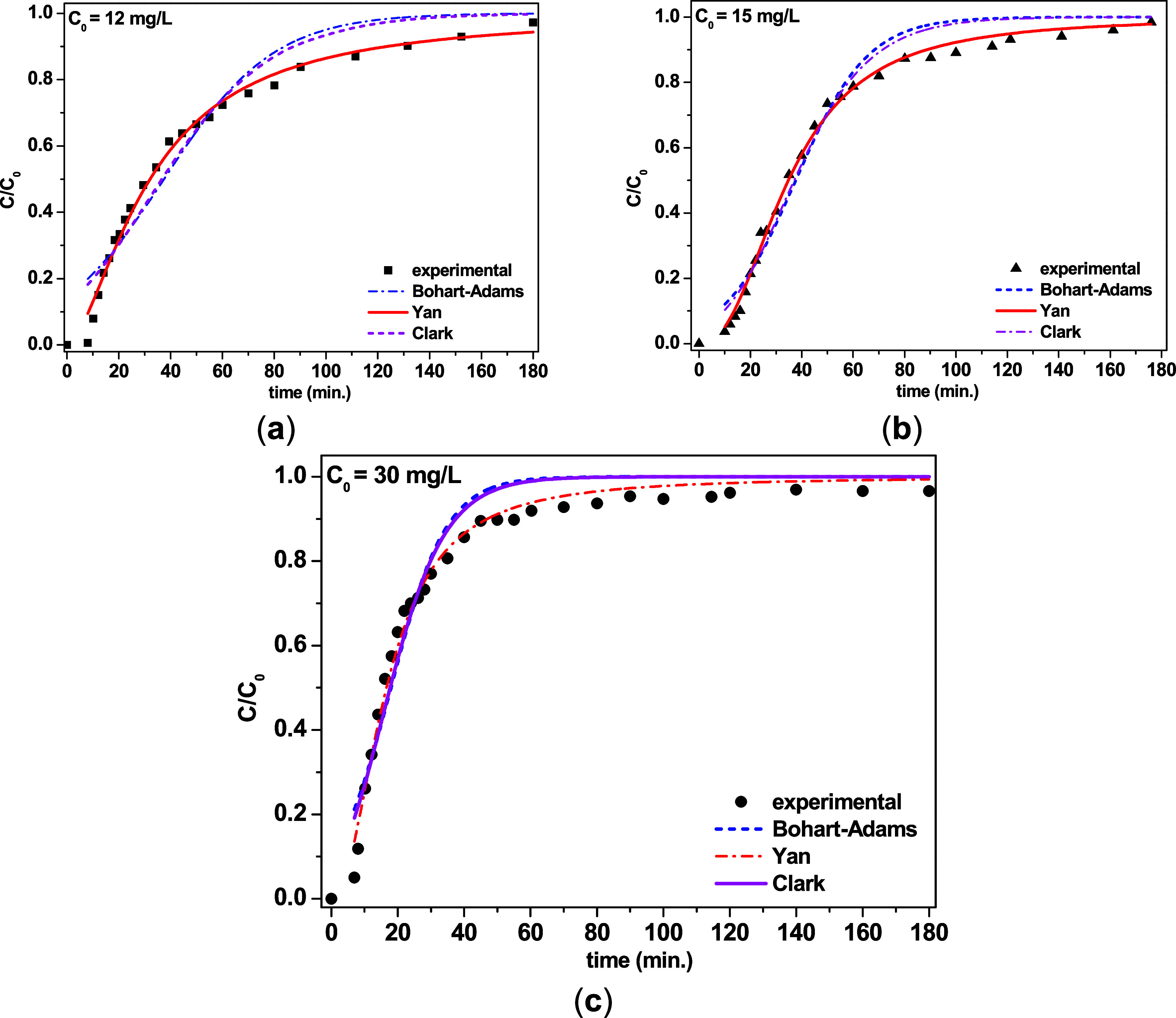
Fixed-bed column adsorption of TC on Al-MCM-41 and fits obtained with the nonlinear Bohart–Adams, Clark, and Yan models; *Q* = 1.5 mL min^–1^, for *C*_0_: (a) 12 mg L^–1^, *W* = 0.0553 g, (b) 15 mg L^–1^, *W* = 0.0561 g, and (c) 30 mg L^–1^, *W* = 0.0560 g.

Nonetheless, the Yan model (*R* ^2^ = 0.996) proved the most effective for describing the entire breakthrough curve, with a predicted adsorption capacity (*q*_Y_) close to the experimental value, making it the most reliable model for representing the behavior of the column under these operating conditions. The Bohart–Adams model was suitable for the initial phase of the process, indicating that surface diffusion was the rate-limiting step in the adsorption process.^33,43,47,48^ The Clark model provided good predictability, especially for intermediate and high concentrations.

Table 6 lists the fitting parameters of the nonlinear equations of the models applied to fixed-bed column experiments. The Bohart–Adams model was able to satisfactorily describe the behavior during the initial adsorption period, with a high *R* ^2^ of 0.965 and a tendency to underestimate the adsorption capacity. The Yan model provided an excellent overall prediction of breakthrough (*R* ^2^ = 0.996), with a predicted *q*_Y_ very close to *q*_exp_, whereas the Clark model (*R* ^2^ = 0.974) showed a good fit, especially at moderate concentrations. These results were consistent with previous work employing other types of adsorbents, showing the practical utility of the Yan model for real applications, while the Bohart–Adams was useful for estimating the initial phase of breakthrough curve, and the Clark model provided good fit to the entire breakthrough curve.^48^

**Table 6:** Parameters of the Nonlinear Bohart–Adams, Yan, and Clark Models for Adsorption of TC by Al-MCM-41 in a Fixed-Bed Column

		Bohart–Adams				Yan			Clark		
*C*_0_ (mg/L)	*q*_exp_ (mg/g)	*q*_BA_ (mg/g)	*k*_BA_ (mL/mg min)	*N*_BA_ (mg/mL)	*R* ^2^	*q*_Y_ (mg/g)	*a*_Y_	*R* ^2^	*r* (1/min)	*A*	*R* ^2^
12	15.7	10.7	3.9	2.0	0.924	10.2	1.63	0.990	0.04	1.67	0.938
15	19.1	15.2	4.5	2.9	0.965	14.3	2.35	0.996	0.06	3.38	0.974
30	18.9	12.7	4.1	2.4	0.938	12.5	2.12	0.987	0.10	2.35	0.948

## 4 Conclusions

The synthesized Al-MCM-41 silica, with a Si/Al ratio of 16.1, exhibited good performance as an adsorbent for the removal of TC from aqueous solutions. Characterization analyses demonstrated that the ordered hexagonal structure and textural properties of the material were maintained after the incorporation of aluminum. Rapid and efficient adsorption was observed in batch studies, with the pseudo-second-order model providing the best fit to the experimental data. The equilibrium isotherms could be satisfactorily described by the Langmuir and Freundlich models, with a high adsorption capacity, consistent with the heterogeneity of the adsorbent surface, related to the presence of aluminum in the silica matrix. The thermodynamic parameters indicated that the adsorption process was spontaneous and exothermic, with possible contributions from physical and chemical interactions, suggesting the existence of mechanisms such as hydrogen bonding, electrostatic interactions, and complexation. Al-MCM-41 showed satisfactory performance in fixed-bed column experiments, with better utilization of the adsorbent bed at high TC concentrations, although the total operating time was shorter. For practical applications and scale-up design, it is recommended to increase the bed height (initial estimate ≈50 mm to reduce the MTZ to ≈30% of the bed) or operate at lower flow rates. The Yan model provided the best description of the adsorption dynamics under continuous flow, while the Clark and Bohart–Adams models were able to describe specific phases of the process. Therefore, the use of Al-MCM-41 is a promising and sustainable strategy for the treatment of effluents containing emerging contaminants, such as antibiotics. Its high efficiency, together with its ability to regenerate and reuse material, can contribute to the reduction of environmental impacts and the development of clean technologies.

## Abbreviations

Al-MCM-41: mobil composition of matter mesoporous silica with aluminum
Si/Al: ratio of silicon–aluminum
TC: tetracycline
FTIR: Fourier transform infrared spectroscopy
TEM: transmission electron microscopy
Δ*H*°: standard enthalpy change
EC: emerging contaminant
ARB: antibiotic-resistant bacteria
ARGs: antibiotic-resistant genes
*t*_b_: breakthrough time
*t*_s_: saturation time
*q*_exp_: maximum adsorption capacity in fixed-column adsorption experiment
FBU: fractional bed utilization
CTMABr: cetyltrimethylammonium bromide
TEOS: tetraethyl orthosilicate
NaOH: sodium hydroxide
NaAlO_2_: sodium aluminate
KBr: potassium bromide
KH_2_PO_4_: potassium dihydrogen phosphate
UV–vis: ultraviolet–visible
*q*_e_: adsorption capacity of the adsorbent at equilibrium
*C*_0_ and *C*_e_: initial and equilibrium concentrations of TC in the liquid phase (mg L^–1^)
*V*: volume of TC solution (mL)
*W*: mass of adsorbent (g of TC)
*q*_max_: maximum adsorption capacity in the batch experiment
*K*_L_: Langmuir isotherm constant
*K*_F_ and *n*: Freundlich constants
*q*_*t*_: adsorption capacity of the adsorbent at time *t*
*k*_1_: pseudo-first-order rate constant
*k*_2_: pseudo-second-order rate constant
*C*_b_: breakthrough concentrations of TC in the liquid phase (mg L^–1^)
*Q*: flow rate
*q*_b_: adsorption capacity of the adsorbent at breakthrough
*q*_s_: adsorption capacity of the adsorbent at saturation
*R* ^2^: determination coefficient
RMSE: root-mean-squared error
*k*_BA_: Bohart–Adams model rate constant (mL min^–1^)
*q*_BA_: maximum adsorption capacity of the Bohart–Adams model
*N*_0_: uptake volumetric capacity (mg L^–1^)
*Z*: bed height (cm)
υ: linear flow rate (cm min^–1^)
*A*: column cross-section area (cm^2^)
*q*_Y_: amount of solute adsorbed of the Yan model
*a*_Y_: constant of the Yan model
*B* and *r*: constants of the Clark model
N_2_: nitrogen gas
*P*/*P*_0_: relative pressure in N_2_ adsorption/desorption isotherms
Δ*G*°: standard Gibbs free-energy change
Δ*S*°: standard entropy change
